# Roles of Enhancer RNAs in RANKL-induced Osteoclast Differentiation Identified by Genome-wide Cap-analysis of Gene Expression using CRISPR/Cas9

**DOI:** 10.1038/s41598-018-25748-3

**Published:** 2018-05-14

**Authors:** Yukako Sakaguchi, Keizo Nishikawa, Shigeto Seno, Hideo Matsuda, Hiroshi Takayanagi, Masaru Ishii

**Affiliations:** 1 Department of Immunology and Cell Biology, Graduate School of Medicine and Frontier Biosciences, Osaka University, 2-2 Yamadaoka, Suita, Osaka, 565-0871 Japan; 2 WPI-Immunology Frontier Research Center, Osaka University, 3-1 Yamadaoka, Suita, Osaka, 565-0871 Japan; 3 Japan Science and Technology Agency, CREST, 5 Sanban-cho, Chiyoda-ku, Tokyo, 102-0075 Japan; 4 Department of Bioinformatic Engineering, Graduate School of Information Science and Technology, Osaka University, 1-5 Yamadaoka, Suita, Osaka, 565-0871 Japan; 5 Department of Immunology, Graduate School of Medicine and Faculty of Medicine, The University of Tokyo, 7-3-1 Hongo, Bunkyo-ku, Tokyo, 113-0033 Japan

## Abstract

Bidirectional transcription has been proposed to play a role associated with enhancer activity. Transcripts called enhancer RNAs (eRNAs) play important roles in gene regulation; however, their roles in osteoclasts are unknown. To analyse eRNAs in osteoclasts comprehensively, we used cap-analysis of gene expression (CAGE) to detect adjacent transcription start sites (TSSs) that were distant from promoters for protein-coding gene expression. When comparing bidirectional TSSs between osteoclast precursors and osteoclasts, we found that bidirectional TSSs were located in the 5′-flanking regions of the Nrp2 and Dcstamp genes. We also detected bidirectional TSSs in the intron region of the Nfatc1 gene. To investigate the role of bidirectional transcription in osteoclasts, we performed loss of function analyses using the CRISPR/Cas9 system. Targeted deletion of the DNA regions between the bidirectional TSSs led to decreased expression of the bidirectional transcripts, as well as the protein-coding RNAs of Nrp2, Dcstamp, and Nfatc1, suggesting that these transcripts act as eRNAs. Furthermore, osteoclast differentiation was impaired by targeted deletion of bidirectional eRNA regions. The combined results show that eRNAs play important roles in osteoclastogenic gene regulation, and may therefore provide novel insights to elucidate the transcriptional mechanisms that control osteoclast differentiation.

## Introduction

Bone-marrow-derived monocyte-macrophage precursor cells (BMMs) differentiate into osteoclasts when stimulated with macrophage-colony-stimulating factor (M-CSF) and the receptor activator of the NF-κB ligand (RANKL)^1^. They are also activated by the cytokines interleukin 17, interferon γ, and tumour necrosis factor α^2^. The RANKL/RANK interaction with M-CSF/c-Fms activates the nuclear factor of activated T cells calcineurin-dependent 1 (Nfatc1)^3–5^, as well as the transcription factors NF-κB^6,7^, c-Fos^8,9^, and JunD^3^. The transcription factors originate downstream of the signalling pathways, and cooperatively facilitate expression of osteoclastogenic genes^10,11^.

Recent studies have identified the expression of various non-coding RNAs during osteoclastogenesis, including circular RNAs, microRNAs, and long non-coding RNAs (lncRNAs)^12^. The genome is pervasively transcribed by a large number of lncRNAs^13–15^, and their expression profiles are tissue specific and alter during various stages of cell differentiation^12^. The length of lncRNA transcripts ranges from 200 base pairs (bp) to 100 kilobase pairs (kbp), and most lncRNAs have a low abundance and lack typical signatures for selective restrictions^12^. Transcripts that originate from regulatory enhancer elements (eRNA) show a distinct signature^3,16,17^. Furthermore, the enhancers can potentially initiate bidirectional RNA synthesis, often in proportion to the transcripts around the transcriptional start site (TSS) of protein-coding RNAs^16^. Recent studies have proposed important roles for the MyoD1 and Snai1 genes^17,18^, the negative elongation factor complex^19^, and 17β-oestradiol (E2)-dependent gene activation as cis-acting enhancer elements^20^.

Nascent eRNAs contain a 7′-methylated cap with a rate of synthesis and levels comparable to neighbouring protein-coding RNAs^16,20,21^. Genome-scale 5′ rapid amplification of cDNA ends (cap analysis of gene expression [CAGE]) to detect TSSs has been used to investigate eRNAs with bidirectional expression patterns^22^. This technique enables global analyses of gene expression from both promoter and eRNA regions.

The function of eRNAs in regulation of gene expression during osteoclast differentiation is still unclear. To identify putative eRNAs involved in osteoclast differentiation, we performed comprehensive gene expression analyses using CAGE to detect bidirectional TSSs with characteristics of enhancer activities during osteoclast differentiation. Using the clustered regularly interspaced short palindromic repeats (CRISPR)/Cas9 system, we identified the eRNA regions of the Nrp2, Dcstamp, and Nfatc1 genes, which regulate protein-coding transcription of these genes, and further identified the roles of these eRNA regions during osteoclast differentiation.

## Results

### Genome-wide identification of TSSs by CAGE in osteoclasts

**Figure 1:**
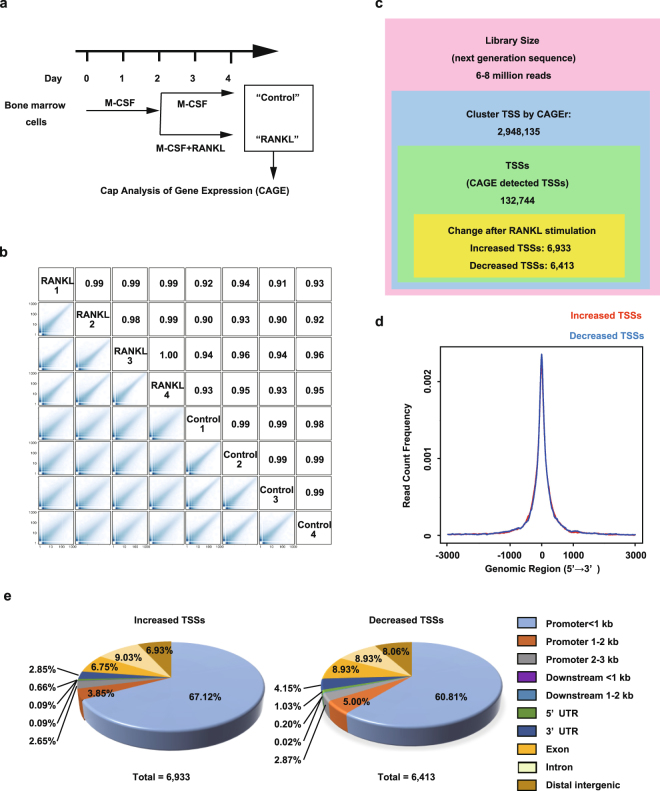
Cap-analysis of gene expression (CAGE) analyses of transcripts during osteoclast differentiation. (**a**) Schematic of the methodology for osteoclast differentiation. (**b**) Pairwise scatter plot of CAGE tag counts per cluster transcription start site (CTSS) and correlations between all possible pairs of samples (four RANKL-stimulated samples and four control samples). Numbers in the boxes represent the values of the correlation coefficients. **(c)** Summary of the pipeline of CAGE transcriptomes for identification of RANKL-induced genes. Among nearly 3 million CTSSs, 132,744 sites were identified as CAGE-detected transcription start sites (TSSs), and 6,933 and 6,413 sites were defined as increased or decreased TSSs, respectively, by differential expression analyses. (**d**) Comparison of the distances between identified TSSs and known promoters. (**e**) Pie charts representing genomic annotations of increased and decreased TSSs.

To identify TSSs that were activated during osteoclast differentiation, we prepared CAGE libraries from BMMs stimulated with or without RANKL (Fig. 1a). We obtained BMMs cultured with M-CSF as osteoclast precursors and stimulated them with RANKL for osteoclasts. In addition, we mapped CAGE tags from samples of RANKL-treated and -untreated (control) BMMs in quadruplicate in the mouse genome (mm10) and identified 2,948,135 cluster TSSs (CTSSs). CAGE tag counts per CTSS showed higher correlations (0.98–1.00) during replication and lower correlations (0.90–0.96) under different conditions, demonstrating the reproducibility of the analyses (Fig. 1b). Next, we clustered and aggregated CAGE tags into a set of 132,744 TSSs. We then performed differential expression analyses between control and RANKL-treated BMMs and found that expression of 6,933 TSSs was significantly increased in RANKL-stimulated BMMs, while that of 6,413 TSSs was significantly decreased (Fig. 1c).

Using transcript-related features from the University of California, Santa Cruz (UCSC) Genome Bioinformatics data resource, the CAGE data identified RANKL-induced TSSs that were usually in known TSS regions (Fig. 1d). We analysed the distribution of RANKL-induced TSSs in important genomic locations such as promoters, downstream sequences of genes, 5′ and 3′ untranslated regions, protein-coding exons, introns, and distal intergenic regions. Approximately 70% of the TSSs were located in proximal promoter regions (within 3,000 bp of known TSSs), 9% in introns, and approximately 6–8% in distal intergenic regions (Fig. 1e). Overall, the CAGE data provided extremely valuable information about genome-wide transcripts during osteoclast differentiation.

### Identification of bidirectional transcripts increased in osteoclasts

**Figure 2:**
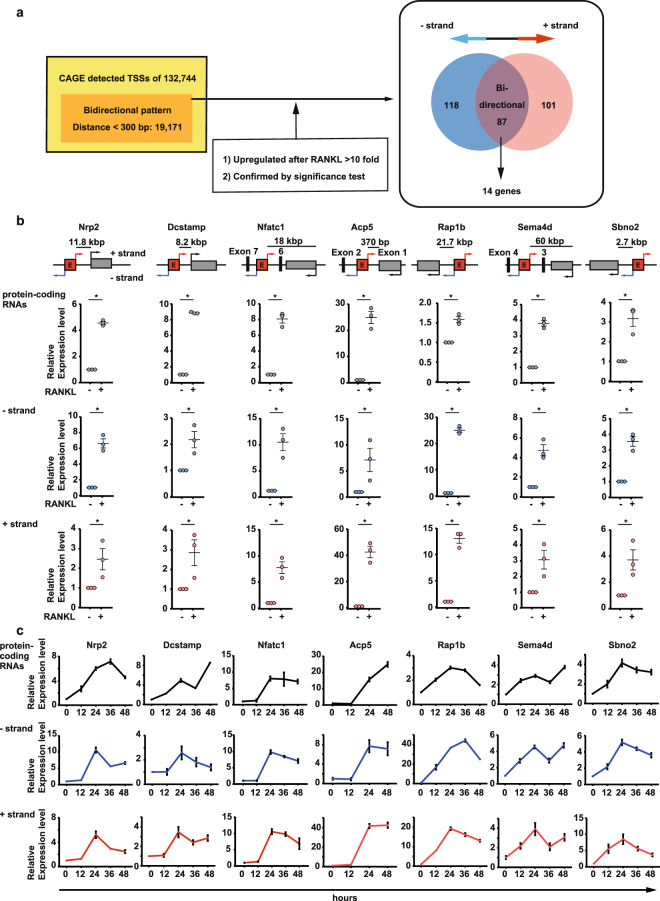
Identification of enhancer RNA (eRNA) candidates induced by RANKL stimulation. (**a**) A summary of the pipeline for bidirectional transcription and the selection of highly altered (>10-fold) RNA levels, confirmed using the significance test. A Venn diagram of bidirectional transcription for the selection of putative eRNA regions associated with osteoclasts. (**b**) Schematic diagram of the genomic loci of Nrp2, Dcstamp, Nfatc1, Acp5, Rap1b, Sema4d, and Sbno2. Quantitative RT-PCR (qRT-PCR) analysis of the relative expression levels of protein-coding RNAs, as well as the − and + strand RNAs following RANKL stimulation for 2 days in RAW 264.7 cells. Data are the mean ± standard error of the mean (SEM); n = 3 biological replicates with 3–4 technical replicates each. *P < 0.05 (Student’s *t*-test) (**c**) Time-course analyses of RANKL-induced alterations in protein-coding RNAs, as well as the − and + strand RNAs of Nrp2, Dcstamp, Nfatc1, Acp5, Rap1b, Sema4d, and Sbno2. Analyses were performed on total RNAs. Data are the mean ± SEM; n = 3 biological replicates with 3–4 technical replicates each.

To identify eRNA candidates, we set a threshold of 300 bp between reverse (−strand) and forward (+strand) TSSs for bidirectional transcribed loci and detected 19,171 sites. After RANKL stimulation, when compared with the control, we made selections based on a difference >10, then confirmed these selections using significant differences. The putative eRNAs showed 188 sites with + strands, 205 sites with −strands, and 87 sites with bidirectional expression. The sites with bidirectional transcription expression were assessed based on location, distance from the protein-coding transcription site, and the predicted permissive enhancer using FANTOM5 analyses and reports associated with osteoclastogenesis (Fig. 2a and Table 1)^23^.

**Table 1:** List of candidate eRNA regions in response to RANKL stimulation.

SYMBOL	^a^annotation	gene strand	^b^distance to TSS	FDR		^c^permissive enhancer (FANTOM5)
				+strand	−strand	
Sbno2	Promoter (2–3 kb)	−	−2769	8.93.E-09	9.12.E-59	−
Jdp2	3′ UTR	+	38448	2.08.E-58	2.09.E-08	−
Nrp2	Distal Intergenic	+	−11851	2.78.E-03	3.39.E-42	−
Egr2	Promoter (2–3 kb)	+	−2178	1.59.E-19	4.39.E-02	−
Nfatc1	Intron	−	51979	8.21.E-10	2.92.E-14	+
Dcstamp	Distal Intergenic	+	−8165	4.62.E-12	3.90.E-03	+
Zbtb7a	Distal Intergenic	+	−7039	4.80.E-12	1.19.E-03	−
Fosl2	Intron	+	−83707	2.00.E-07	3.40.E-10	+
Zbtb7a	Intron	+	5049	1.66.E-08	1.31.E-03	−
Nrp2	Distal Intergenic	+	−7217	3.64.E-08	1.44.E-02	−
Rap1b	Distal Intergenic	−	−21278	2.00.E-07	1.94.E-04	+
Acp5	Promoter (<=1 kb)	−	370	5.44.E-07	6.07.E-02	−
Fos	Distal Intergenic	+	36547	3.33.E-04	2.14.E-05	−
Sema4d	Promoter (<=1 kb)	−	579	6.92.E-05	2.11.E-02	+
Rnf19b	Intron	+	9719	3.50.E-04	1.88.E-02	−
Nrp2	Distal Intergenic	+	−24014	1.01.E-03	3.62.E-02	−
Ltbp1	Intron	+	93668	2.11.E-02	4.15.E-02	−
Fos	Distal Intergenic	+	12780	3.22.E-02	3.91.E-02	−

^**a**^Annotation of gene segments defined by edgeR.

^**b**^The distance to the transcription start site (TSS) measured from the TSS of protein-coding measured by edgeR.

^**c**^The enhancer regions were classified into robust and permissive, based on the read counts. The permissive enhancer was defined by FANTOM5 mouse permissive enhancer phase 1 and 2^23^.

Using quantitative reverse transcription-polymerase chain reaction (qRT-PCR), we confirmed RANKL-induced bidirectional transcription expression in RAW 264.7 cells. Among the 87 sites, we first selected seven sites based on previous reports^3–5,10,24,25^ in association with osteoclasts. These bidirectional transcripts increased in RAW 264.7 cells following RANKL stimulation, and were usually generated at the 5′-flanking regions of the Nrp2, Dcstamp, Rap1b and Sbno2 genes. In contrast, the bidirectional transcripts of Nfatc1, Acp5 and Sema4d were located in introns (Fig. 2b). In time-course studies following RANKL stimulation, gene expression levels increased in proportion to the increase in bidirectional transcription expression of the + and − strands (Fig. 2c). Protein-coding RNA levels were measured by qRT-PCR using primers unrelated to the putative eRNA sequences. The results showed that the RANKL-dependent increase in expression of these seven transcripts was accompanied by bidirectional transcription in RAW 264.7 cells. The bidirectional transcripts increased at the same time as the expression of the protein-coding RNAs after RANKL stimulation. These results imply that these regions act as eRNAs in the regulation of protein-coding RNA expression.

### eRNAs are necessary for induction of protein-coding RNAs

**Figure 3:**
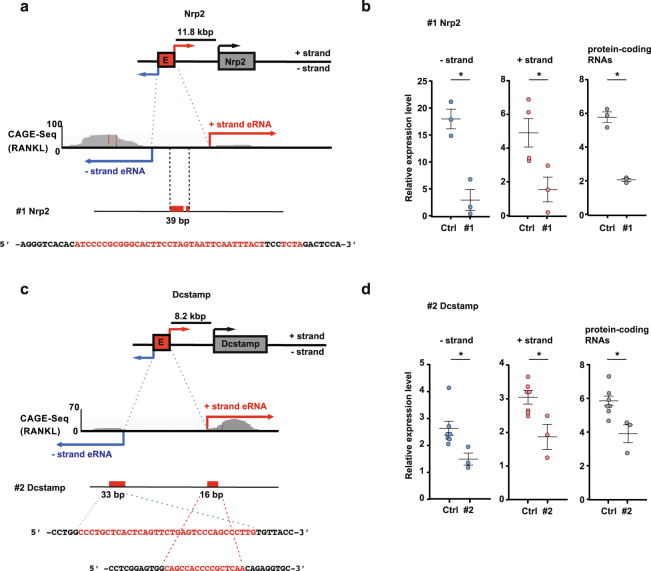
The effect of deletions in the 5′ putative eRNA regions of Nrp2 and Dcstamp using CRISPR/Cas9. (**a**,**c**) Schematic diagram of the genomic loci with the CAGE profile and the deleted regions (deletions are marked with red letters) in RAW 264.7 cells. **(b**,**d**) qRT-PCR analysis of the relative expression levels of protein-coding RNAs as well as the putative − and + strand eRNAs following RANKL stimulation of the #1 Nrp2 putative eRNA region-deletion mutant and the targeted deletion of the #2 Dcstamp mutant. Control cells (Ctrl) were transfected with the scrambled sequence shown in Supplementary Table S3. Data denote the mean ± SEM from independent biological replicates; #1 Nrp2 (n = 3), Ctrl (Nrp2, n = 3–4), #2 Dcstamp (n = 3), Ctrl (Dcstamp, n = 7) with 3–4 technical replicates each. Analyses were performed on total RNAs. *P < 0.05 [Student’s *t*-test for (**b**); Dunnett’s test for (**d**) to evaluate with other clone, #3 and 4. The results of #3 and 4 are shown in Supplementary Figure S2].

To investigate the role of eRNAs, we deleted eRNA regions in RAW 264.7 cells using the CRISPR/Cas9 genome editing system^26^ (Supplementary Figure S1). The schema shows that single cells were sorted and expanded to obtain DNA for sequencing. In the putative Nrp2 eRNA region of approximately 200 bp, we deleted the 39 bp eRNA regions for bidirectional transcription (#1 Nrp2) (Fig. 3a). The #1 Nrp2 mutant cells showed marked decreases in both eRNA and Nrp2 protein-coding RNA levels (Fig. 3b).

The role of the putative eRNA region for the Dcstamp gene was examined by partial deletion mutants. We obtained three deletion mutant clones. One clone (#2 Dcstamp), which had deletions at both the − and + strand regions, showed a significant decrease in bidirectional transcript expression and a decrease in protein-coding Dcstamp RNA level (Fig. 3c). Clone #3 Dcstamp, deleted at the − strand RNA region, and clone #4 Dcstamp, deleted at the + strand RNA region, showed similar effects (Supplementary Figures S2a,b). These effects on the protein-coding RNAs showed that the bidirectional transcripts play a role as eRNA during osteoclast differentiation.

### The role of the intronic putative eRNA in Nfatc1 gene expression

**Figure 4:**
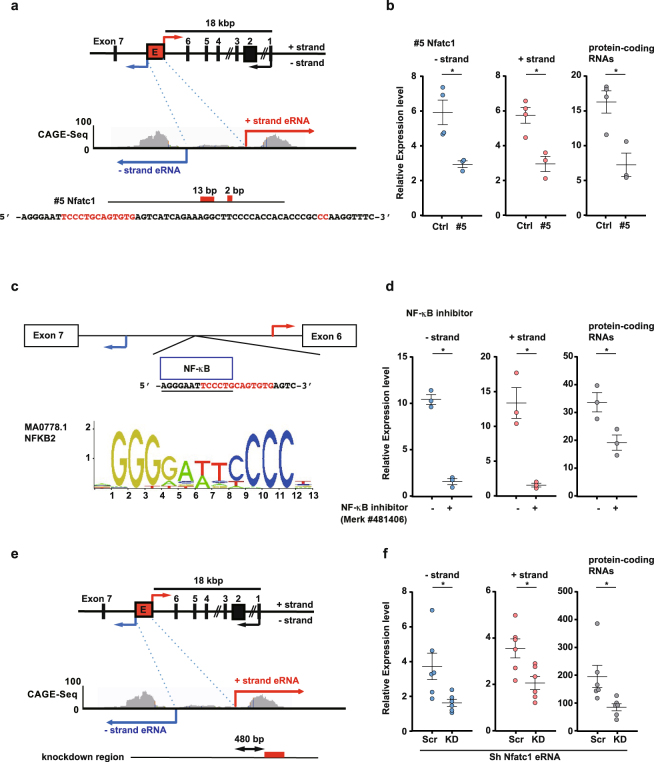
The effect of intronic putative eRNAs on Nfatc1 protein-coding RNA expression. (**a**) The effect of deletions in the intronic putative eRNA region of Nfatc1 using CRISPR/Cas9. Schematic diagram of the genomic locus and the deleted regions (deletions are marked with red letters) in RAW 264.7 cells. (**b**) qRT-PCR analysis of the relative expression levels of protein-coding RNAs as well as the − and + strand eRNAs following RANKL stimulation. Data are the mean ± SEM; n = 3–4 biological replicates with 3–4 technical replicates each. Ctrl cells were transfected with the scrambled sequence shown in Supplementary Table S3. (**c**) Motif analyses of the deleted eRNA region. The high score motif (NF-κB) of the deleted sequence was analysed using JASPAR. **(d)** The effect of the NF-κB inhibitor (Merck #481406) on eRNA levels in RAW 264.7 cells. Data are the mean ± SEM; n = 3 biological replicates with 3–4 technical replicates each. **(e)** Suppression of the + strand eRNA by small hairpin (shRNA) transfection. Schematic of the genomic locus with the CAGE profile and the deleted regions in monocyte-macrophage precursor cells (BMMs). (**f**) qRT-PCR analysis of the relative expression levels of protein-coding RNAs, as well as the − and + strand eRNAs following RANKL stimulation in shRNA-transfected BMMs. Analyses were performed on total RNAs. Data denote the mean ± SEM; n = 6 biological replicates with 3–4 technical replicates each. *P < 0.05; NS, not significant [Student’s *t*-test for (**b**) and (**d**); Dunnett’s test for **(f)**].

Enhancer regions have been detected not only in the 5′ flanking region but also in other genomic regions, including intronic regions^22^. Because we identified TSSs in the genomic region between exons 6 and 7 of the Nfatc1 gene, we investigated the function of intronic putative eRNA by generating a knockout of the putative intronic eRNA region of the Nfatc1 gene (#5 Nfatc1) (Fig. 4a). #5 Nfatc1 cells showed a marked decrease in the levels of intronic putative eRNAs, demonstrating the role of the intronic eRNA region in bidirectional transcription (Fig. 4b). The #5 Nfatc1 mutant had two separate site deletions of 13 bp and 2 bp (Fig. 4a; deletions are designated by red letters). Using JASPAR software, it was determined that the DNA sequence in the eRNA regions contained a motif for the transcription factor, NF-κB (Fig. 4c). To determine the function of the intronic eRNA region, we next determined whether an NF-κB inhibitor^7^ would suppress bidirectional transcription. Similar to the results obtained for the #5 Nfatc1 cells, treatment with NF-κB inhibitor decreased the expression level of bidirectional transcripts generated from the putative intronic eRNA region of the Nfatc1 gene (Fig. 4d), similar to the decrease in Nfatc1 protein-coding RNA level following NF-κB inhibitor treatment.

To confirm the role of putative intronic eRNAs, we prepared a retroviral vector construct containing small hairpin RNA (shRNA) against the + strand of the Nfatc1 intronic transcript (Fig. 4e). Knockdown by shRNA showed decreased expression of bidirectional transcripts and Nfatc1 protein-coding RNA, compared to the scrambled control shRNA (Fig. 4f). Collectively, the results imply that intronic Nfatc1 eRNA is functionally important for RANKL-induced transcription of the Nfatc1 gene.

### eRNA is required for osteoclast differentiation

**Figure 5:**
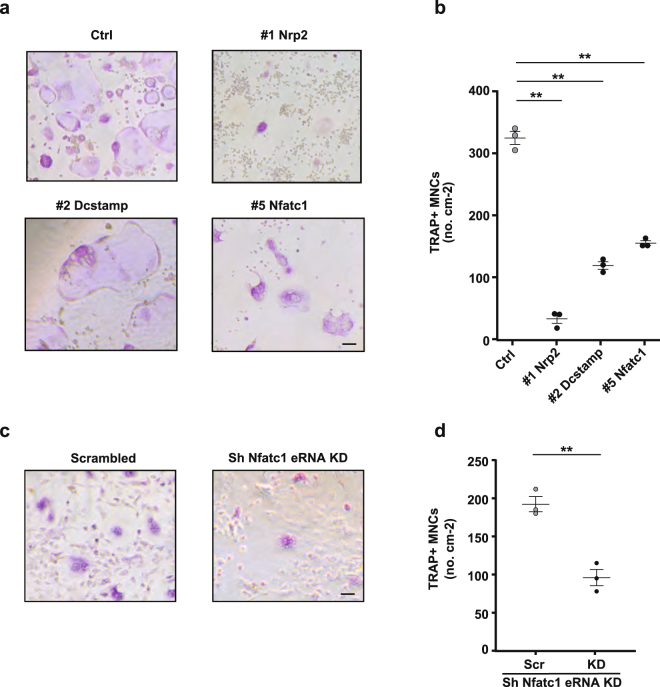
The effect of eRNA on osteoclast differentiation. (**a**) Tartrate-resistant acid phosphatase (TRAP)-stained cells showing osteoclast differentiation in control and knockout cells. (**b**,**d**) The numbers of TRAP-positive cells with more than three nuclei were counted. **(c)** TRAP-stained cells showing osteoclast differentiation mediated-knockdown with sh-scrambled (Scr) and sh-Nfatc1 eRNA as in Fig. 4d. Scale bars, 100 μm. Data denote the mean ± SEM from three independent biological replicates (n = 3) for each group. **P < 0.01 [Dunnett’s test for (**b**) together with #3 and 4 Dcstamp mutants; the rest of the results are shown in Supplementary Figure S2d; Student’s *t*-test for (**d**)]. The experiments were carried out three times.

To investigate the role of eRNAs in osteoclast differentiation further, we evaluated osteoclast differentiation *in vitro* following RANKL stimulation by counting multinucleated cells (MNCs) positive for the osteoclast marker tartrate-resistant acid phosphatase (TRAP). RAW 264.7 cells or BMMs were cultured for 3 days with RANKL^27^. Compared to control cells, the cells with eRNA-region deletions of Nrp2, Dcstamp, and Nfatc1 showed remarkably impaired formation of TRAP‐positive MNCs (Fig. 5a,b and Supplementary Figures S2c and 2d). Knockdown of Nfatc1 intronic eRNA also decreased TRAP-positive cells compared to the scrambled shRNA control cells (Fig. 5c,d). These results imply that expression of the eRNAs for the Nrp2, Dcstamp, and Nfatc1 genes positively regulates osteoclast differentiation.

## Discussion

Cell-stage-dependent eRNAs with highly dynamic and transient expression may play critical roles in the differentiation of various cells^18,20^. Here, we investigated the eRNA regions that generate bidirectional transcription in RANKL-stimulated BMMs. CAGE data showed that the 5′ eRNA regions of the Nrp2 and Dcstamp genes were distantly located from the annotated promoter regions and that the intronic eRNA regions were located between exons 6 and 7 of the Nfatc1 gene.

Enhancers were initially described as short DNA regions with the ability to drive target gene expression independently of the genomic distance and orientation of the gene promoters. Enhancers were defined by their hypersensitivity to DNase treatment, ability to bind transcription factors, and their epigenomic markers. Annotated putative enhancers amount to an extremely large number in humans (>400,000 to ~1 million). A large-scale transcriptome profile defined by CAGE analyses showed that enhancers associated with transcriptional activity still yielded an abundant number of non-coding RNAs in humans (40,000–65,000)^22^. eRNAs generally display low stability and abundance^22^; thus, an early report hypothesized that non-coding transcripts including eRNAs are transcriptional noise, and this hypothesis has been examined for many eRNAs^22,28^. Enhancers often generate bidirectional transcripts and functional interaction mediated by the formation of enhancer–promoter looping^29,30^. Our study initially clarified the functions of candidate eRNAs by identifying their roles in transcriptional enhancer activities in cognate gene control and cell differentiation, while characterization of their mechanisms of action remains for a later study.

Several models of the functions of eRNAs have been proposed. eRNA may regulate the chromatin accessibility of target promoters and RNA polymerase II binding, although the mechanism of the functional interaction of widely separated enhancers and promoters remains unclear. Alternatively, the enhancer–promoter interaction could be a consequence rather than a cause. A recent report proposed a simple mechanism for eRNA called “transcription factor trapping”, which involved a general role for a large group of eRNAs in facilitating binding of transcription factors^31^. eRNA has generally been analysed in terms of *cis*-acting effects on target genes, but several reports have demonstrated the effects of eRNAs on the expression of many genes, including those on other chromosomes^32^. In addition, eRNAs and transcribing enhancers may affect the target gene, based on the spatial proximity of the three dimensional genome^33^.

The role of the intronic enhancer may vary, depending on enhancer or repressor activity over the coding transcripts. It may also depend on splicing events. Recent reports have shown that two intronic transcribed enhancers modulate the isoform decision of overlapping sense-coding genes by transcription interference^34^. However, our study showed that the intronic enhancer plays a crucial role in enhancing Nfatc1 transcription during RANKL-induced osteoclast differentiation. #5 Nfatc1 cells, which lack the function of the enhancer region located in the intronic region of the Nfatc1 gene, would provide a suitable genetic model to study how this results in local enhancer activity, and whether this activity has a global effect.

The CRISPR/Cas9 system has been used to identify endogenous enhancer elements and to characterise domains essential for their activity^35^. Here, we applied the CRISPR/Cas9 system to characterise the functions of eRNA regions in the expression of protein-coding RNAs associated with osteoclast differentiation. Nfatc1 plays a pivotal role in osteoclast activation through induction of β3 integrin and c-src, and via upregulation of various genes in a series of differentiation processes^36^. The 18-bp deletion in the eRNA region in #5 Nfatc1 mutant cells shows the importance of this region in the expression of bidirectional eRNAs and Nfatc1 protein-coding RNA. Moreover, this mutant showed impaired osteoclastogenic differentiation, as measured using TRAP staining. NF-κB inhibition also suppressed bidirectional transcription from this eRNA region, indicating the role of NF-κB-mediated signalling in regulation of intronic enhancer activity.

The eRNA regions for the Nrp2, Dcstamp, and Nfatc1 genes play similar roles as enhancers for protein-coding RNA expression, and deletions of their partial sequences impaired osteoclast differentiation. This implies that the RANKL-induced signalling network interacts with eRNA expression.

In conclusion, RANKL-induced eRNAs were functionally active in enhancing expression of protein-coding RNAs, which play important roles during osteoclast differentiation. These results emphasise the involvement of non-coding eRNAs in regulation of functional genes via an orchestrated mechanism during cell differentiation.

## Material and Methods

### Cell culture

*In vitro* osteoclast differentiation has been described previously^27,37^. C57BL/6J mice were purchased from CLEA (Tokyo, Japan). The animal protocols were approved by the Institutional Animal Care and Use Committee of Osaka University, and all experiments were performed in accordance with the relevant guidelines and regulations. Briefly, bone-marrow-derived cells from C57BL/6J mice cultured with M-CSF (10 ng/mL) for 2 days were used as osteoclast precursors, and were further cultured with RANKL (50 ng/mL) in the presence of M-CSF (10 ng/mL) for 2 days to obtain osteoclasts. Total RNAs were prepared using an RNeasy Mini Kit (Qiagen, Hilden, Germany) and subjected to CAGE analyses. RAW 264.7 cells obtained from the American Type Culture Collection (Rockville, MD, USA) were cultured in α-minimal essential medium containing 10% foetal bovine serum and 1% penicillin-streptomycin solution. The NF-κB inhibitor was Merck #481406.

### RNA isolation and qRT-PCR analyses

Total RNA and cDNA were prepared using the RNeasy Mini Kit and Superscript III reverse transcriptase (Invitrogen, Carlsbad, CA, USA) according to the manufacturers’ instructions. qRT-PCR was performed using a TP800 Thermal Cycler Dice Real Time System (TaKaRa, Shiga, Japan). The expression of every sample was calculated relative to that of the β-actin housekeeping gene. The primer sequences are listed in Supplementary Tables S1 and S2.

### TRAP staining

For TRAP staining^38^, RAW 264.7 cells were cultured in a 48-well plate at a density of 1 × 10^4^ cells/well, and BMMs were cultured in a 48-well plate at a density of 2 × 10^4^ cells/well. Three days after RANKL stimulation, the cells were fixed in 4% paraformaldehyde for 15 min and stained using an acid phosphatase, leukocyte (TRAP) kit (Sigma-Aldrich, St. Louis, MO, USA). The numbers of TRAP-positive and multinucleated cells (>3 nuclei) were counted.

### Vector preparation for the CRISPR/Cas9 system

pX330-U6-Chimeric_BB-CBh-hSpCas9 was purchased from Addgene, (Cambridge, MA, USA). The single guide RNA sequences targeting the Nrp2, Dcstamp, and Nfatc1 eRNA regions were designed using Optimized CRISPR Design^39^. The guide sequences are listed in Supplementary Table S3. The universal negative control containing a scrambled sequence was 5′-GCACTACCAGAGCTAACTCA-3′^40^. The targeting vector of each eRNA region was transfected together with the pmaxGFP^®^ vector (Lonza, Basel, Switzerland) into RAW 264.7 cells using the Amaxa Cell Line Nucleofector Kit V (Lonza). The transfected cells were cultured for 2 days, and single-cell sorted using an SH800 cell sorter (Sony, Tokyo, Japan). The knockout clones used in these studies were validated by sequencing.

### Retroviral gene transfer

The retroviral vector, pSIREN-shNfatc1 eRNA, was constructed by inserting annealed oligonucleotides into RNAi-Ready pSIREN-RetroQ (BD Biosciences, San Jose, CA, USA). The oligonucleotide sequences are listed in Supplementary Table S4. The pSIREN-shControl was constructed as previously described^27^. Retroviral packaging was performed by transfecting the plasmids into Plat-E cells using FuGENE^®^ 6 Transfection Reagent (Promega, Madison, WI, USA) as previously described^41^. Ten hours after inoculation with retroviruses, BMMs were stimulated with RANKL (50 ng/mL) for 2 days.

### CAGE library preparation and data processing

CAGE libraries were prepared from 5 µg of total RNA purified from primary cells stimulated with RANKL (four samples) or without RANKL (control, four samples). We used the protocol for the construction of no-amplification non-tagging CAGE libraries for Illumina sequencers (nAnT-iCAGE)^42^. Prior to sequencing eight libraries, different barcodes were pooled and applied to the same sequencing lane. The resulting 8-plex nAnT-iCAGE libraries were sequenced using single end reads of 50 bp using HiSeq 2500^®^ (Illumina, San Diego, CA, USA). CAGE tags were mapped to the mouse genome (mm10) using Bowtie2 version 2.2.5^43^. CTSSs, which corresponded to promoter candidates, were identified using the Bioconductor package, CAGEr, version 1.14.0^44^ and BSgenome.Mmusculus.UCSC.mm10, version 1.4.0^45^. CAGE tag counts per CTSS were plotted as scatter plots and Pearson correlations between all possible pairs of samples were calculated. To compare genome-wide transcriptional activities across samples, individual CTSSs were clustered into tag clusters from all eight samples into a single set of TSSs (non-overlapping consensus clusters). The CAGE data have been deposited in the Sequence Read Archive (SRA) database (accession numbers, SRP137592 and SRP137597).

### Differential expression analyses of CAGE

Differential expression analyses were performed using the Bioconductor package, edgeR, version 3.16.5^46^. Differentially expressed TSSs were defined as different promoters with a false discovery rate <0.1 between unpaired samples derived from corresponding samples (four controls and four RANKL-stimulated samples). Differentially expressed TSSs were divided into increased and decreased TSS groups by their fold-change values. Calculations of distances from identified TSSs to known promoters and genomic feature annotations were performed using the Bioconductor package, ChIPseeker, version 1.6.7^47^ with TxDb.Mmusculus.UCSC.mm10.knownGene, version 3.2.2^48^.

### Statistical analyses

Statistical analyses were performed using Student’s *t*-test for comparisons between two groups, and Dunnett’s test for multiple comparisons using JMP Pro 12.0 (SAS, Cary, NC, USA). All data were expressed as means ± standard error of the mean, and the statistical validation is shown in each figure legend. The results are representative examples of three or more independent experiments. The indicated replicates are biological.

## Electronic supplementary material

Supplementary information
