# Supplementary material for: Roles of Enhancer RNAs in RANKL-induced Osteoclast Differentiation Identified by Genome-wide Cap-analysis of Gene Expression using CRISPR/Cas9
Source: Sci Rep. 2018 May 14;8:7504. doi: 10.1038/s41598-018-25748-3 (PMC5951944; doi:10.1038/s41598-018-25748-3)
Supplement: Supplementary file 1 — Supplementary information [file 41598_2018_25748_MOESM1_ESM.pdf]

Supplementary Information

Roles of Enhancer RNAs in RANKL-induced Osteoclast Differentiation Identified by Genome-wide Cap-analysis of Gene Expression using CRISPR/Cas9

Yukako Sakaguchi[1,2,3], Keizo Nishikawa[1,2,3], Shigeto Seno[4], Hideo Matsuda[4], Hiroshi Takayanagi[5] and Masaru Ishii[1,2,3]

[1]*Department of Immunology and Cell Biology, Graduate School of Medicine and Frontier Biosciences, Osaka University, 2-2 Yamadaoka, Suita, Osaka 565-0871, Japan.*

[2]*WPI-Immunology Frontier Research Center, Osaka University, 3-1 Yamadaoka, Suita, Osaka 565-0871, Japan.*

[3]*Japan Science and Technology Agency, CREST, 5 Sanban-cho, Chiyoda-ku, Tokyo 102-0075, Japan.*

[4]*Department of Bioinformatic Engineering, Graduate School of Information Science and Technology, Osaka University, 1-5 Yamadaoka, Suita, Osaka 565-0871, Japan.*

[5]*Department of Immunology, Graduate School of Medicine and Faculty of Medicine, The University of Tokyo, 7-3-1 Hongo, Bunkyo-ku, Tokyo 113-0033, Japan.*

Corresponding authors:

Address correspondence to Keizo Nishikawa (nishi@ifrec.osaka-u.ac.jp) and Masaru Ishii (mishii@icb.med.osaka-u.ac.jp)

Supplementary Table S1: List of qRT-PCR protein-coding gene primers

	Sequence (5'-3')	
Name	Forward	Reverse
Actb	CTTCTACAATGAGCTGCGTG	TCATGAGGTAGTCTGTCAGG
Nrp2	CCACCAGAACTGTGAGTGGA	CAATCTTCTGGTTGGGTTCG
Dcstamp	TCCTCCATGAACAAACAGTTCCAA	AGACGTGGTTTAGGAATGCAGCTC
Nfatc1	TGCTCCTCCTCCTGCTGCTC	CGTCTTCCACCTCCACGTCG
Acp5	CGTCTCTGCACAGATTGCAT	AACTGCTTTTTGAGCCAGGA
Rap1b	CCAACAGGGAGCCACAGTATTT	GGCCACCTCAAAGTCGTTGT
Sema4d	CCAGATAGTGGTAGACAGGACC	GTCTCCTCGATGACATGCACCT
Sbno2	AGACATCCCAGACACACCTG	TGAGAAGTGGAGTGCTGGAG

Supplementary Table S2: List of qRT-PCR eRNA primers

		Sequence (5'-3')	
Name	Strand	Forward	Reverse
Nrp2	−	CTACGAGATGCACAGTCTCC	CGGCCTGGTCACCTTGGACC
	+	GTAGCAGGCTGGCTACCAAG	CCAAAAGGGCACATACTAAAG
Dcstamp	−	TACTGTCACCCCATGCACCT	AGAGGTCACAGGAGGAAAAGG
	+	GCATGTGAGCAGACAGGAAA	CAAGCCTCTTCTCTGCCATT
Nfatc1	−	TGAACTGGAGCACAGTTTCCT	CCCTCCTCCCTGCTCAGA
	+	CCCATCAGTGTGAGGCACT	CAAAGCTGCCCTCCTAACCT
Acp5	−	ACTCGGAGACAGTCCCACAC	CATGTAACAGCCCCCTGTCT
	+	TCCTTGAACACTCACCACCA	ACGGAACGAGAGATTCCAGA
Rap1b	−	CACGTCAGCACTTCCTGGTA	GAATCTTGGAACACCGTTGC
	+	CATCCCTGGACTTTTCTGGA	CAGGAGGGCAGAGGTAGAGTT
Sema4d	−	GCCAGCCGTACTACACCTTC	CCAAATCCCGTGTAGAGAGC
	+	GCACGGTGATGTAGGACACA	ACCTGTGTGTTCATGGCTCT
Sbno2	−	CTGGTGCACCTGCCTGAT	AGCTAGCTTCCGTCCAGGTT
	+	TATGGAAGGGGACACTGAGG	TATATCCTTTGCGGCTGCTC

Supplementary Table S3: List of sgRNA primers

	Sequence (5'-3')	
Name	Forward	Reverse
Scramble	GCACTACCAGAGCTAACTCA	TGAGTTAGCTCTGGTAGTGC
Nrp2_1	GACCAGGGTCACACATCCCC	GGGGATGTGTGACCCTGGTC
Nrp2_2	GCTTTAGCAGGTGGAGTCTA	TAGACTCCACCTGCTAAAGC
Dcstamp_1	GCAGGCACCTCTGTTGAGCG	CGCTCAACAGAGGTGCCTGC
Dcstamp_2	GAAGAGGTCATGGGACAGAG	CTCTGTCCCATGACCTCTTC
Dcstamp_3	CTGCTGGGTAACACAAGGGC	GCCCTTGTGTTACCCAGCAG
Nfatc1_2-1	TTCTGATGACTCACACTGCA	TGCAGTGTGAGTCATCAGAA
Nfatc1_2-2	TTCCCCACCACACCCGCCCA	TGGGCGGGTGTGGTGGGGAA

Supplementary Table S4: List of shRNA primers

Name		Sequence (5'-3')
Nfatc1 +strand	Forward	CCACCAGAACTGTGAGTGGATTAGGCTTATCCACTCACAGTTCTGGTGGTTTTTG
	Reverse	CAAAAACCACCAGAACTGTGAGTGGATAAGCCTAATCCACTCACAGTTCTGGTGG

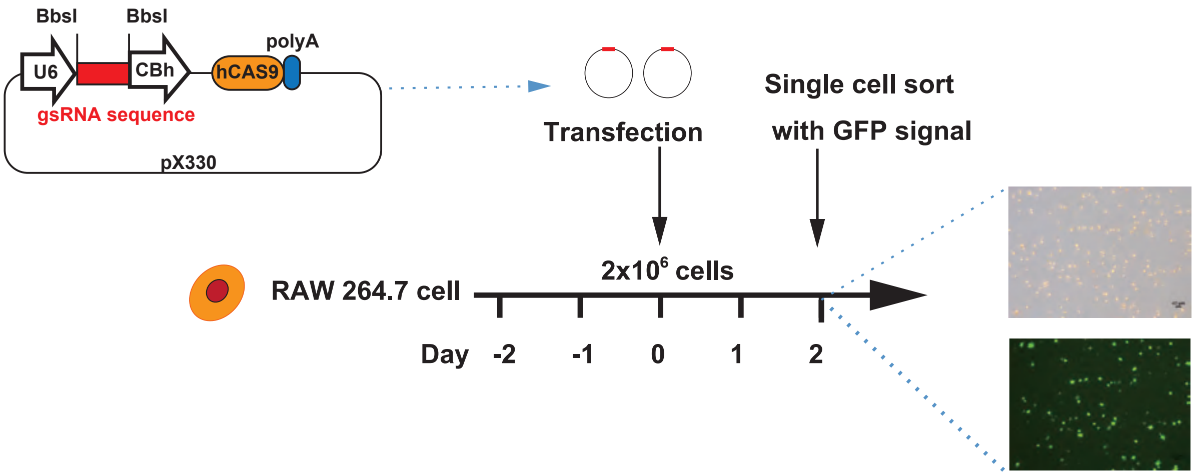

Supplementary Figure S1: Schematic for the production of mutant RAW 264.7 cells using the CRISPR/Cas9 system. The knockout vector with a single guide RNA sequence in pX330 -U6-Chimeric_BB-CBh-hSpCas9 was transfected together with the pmaxGFP vector into RAW 264.7 cells. After 2 days, green-fluorescent-protein-positive cells were single-cell sorted to establish mutant clones. Clones with deletions in the eRNA regions were determined by DNA sequencing.

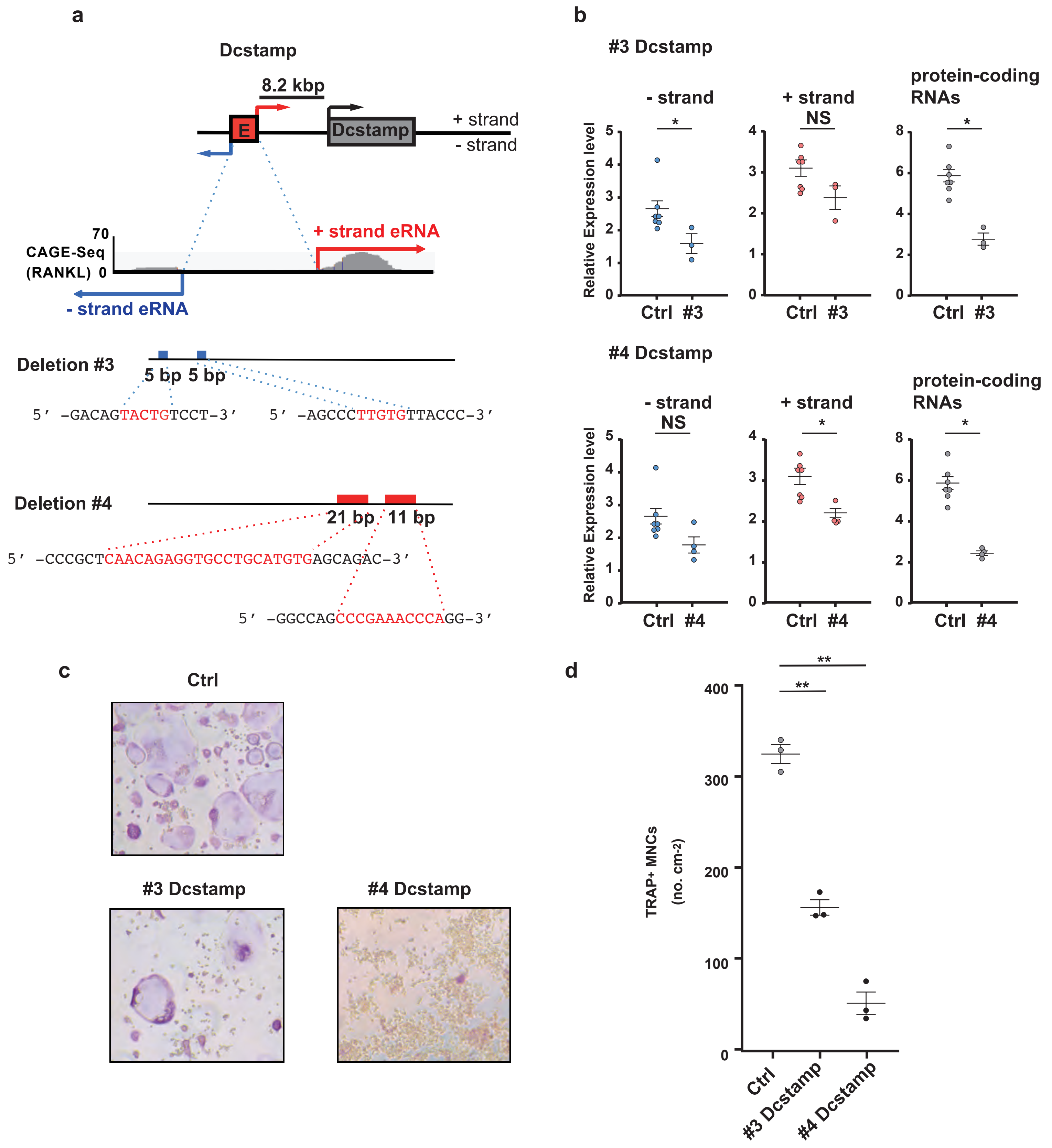

Supplementary Figure S2: The effect of deletions in the 5’ eRNA region of Dcstamp. (a) A diagram of the Dcstamp genomic locus using the CRISPR/Cas9 system as in Figure 3. The sequences of the deleted genomic regions of #3 and #4 Dcstamp cells are denoted with dashes in comparison with the wild-type DNA sequence. (b) qRT-PCR analysis of the relative expression of the protein-coding RNAs, as well as the − and + strand eRNAs in mutant #3 and #4 Dcstamp cells following RANKL stimulation compared with Ctrl cells transfected with a scrambled sequence vector. Analyses were performed on total RNAs. Data denote the mean ± SEM from independent biological replicates; #3 and #4 (n = 3-4); Ctrl (n = 7) with 3-4 technical replicates each. Statistical analysis was carried out by Dunnett’ s test in Figure 3d and the rest of the results are shown here. *P < 0.05. (c) In vitro osteoclast differentiation. TRAP-positive cells were counted in #3 and #4 Dcstamp cells, and shown in the panel (d). Statistiacal analysis for (d) was performed by Dunnett’ s test in Figure 5b and the rest of the results are shown here. Data denote the mean ± SEM from three independent biological replicates (n = 3) for each group. **P <0.01.

.
